# Is reporting on interventions a weak link in understanding how and why they work? A preliminary exploration using community heart health exemplars

**DOI:** 10.1186/1748-5908-3-27

**Published:** 2008-05-20

**Authors:** Barbara L Riley, JoAnne MacDonald, Omaima Mansi, Anita Kothari, Donna Kurtz, Linda I vonTettenborn, Nancy C Edwards

**Affiliations:** 1 Centre for Behavioural Research and Program Evaluation, University of Waterloo, Waterloo, Ontario, Canada; 2 School of Nursing, University of Ottawa, Ottawa, Ontario, Canada; 3 School of Nursing, McGill University, Montreal, Quebec, Canada; 4 Bachelor of Health Sciences Program, University of Western Ontario, London, Ontario, Canada; 5 School of Nursing, University of British Columbia Okanagan, Kelowna, British Columbia, Canada; 6 Bachelor of Science in Nursing Program, Faculty of Health Sciences, Douglas College, New Westminster, British Columbia, Canada; 7 Department of Epidemiology and Community Medicine, University of Ottawa, Ottawa, Ontario, Canada

## Abstract

**Background:**

The persistent gap between research and practice compromises the impact of multi-level and multi-strategy community health interventions. Part of the problem is a limited understanding of how and why interventions produce change in population health outcomes. Systematic investigation of these intervention processes across studies requires sufficient reporting about interventions. Guided by a set of best processes related to the design, implementation, and evaluation of community health interventions, this article presents preliminary findings of intervention reporting in the published literature using community heart health exemplars as case examples.

**Methods:**

The process to assess intervention reporting involved three steps: selection of a sample of community health intervention studies and their publications; development of a data extraction tool; and data extraction from the publications. Publications from three well-resourced community heart health exemplars were included in the study: the North Karelia Project, the Minnesota Heart Health Program, and Heartbeat Wales.

**Results:**

Results are organized according to six themes that reflect best intervention processes: integrating theory, creating synergy, achieving adequate implementation, creating enabling structures and conditions, modifying interventions during implementation, and facilitating sustainability. In the publications for the three heart health programs, reporting on the intervention processes was variable across studies and across processes.

**Conclusion:**

Study findings suggest that limited reporting on intervention processes is a weak link in research on multiple intervention programs in community health. While it would be premature to generalize these results to other programs, important next steps will be to develop a standard tool to guide systematic reporting of multiple intervention programs, and to explore reasons for limited reporting on intervention processes. It is our contention that a shift to more inclusive reporting of intervention processes would help lead to a better understanding of successful or unsuccessful features of multi-strategy and multi-level interventions, and thereby improve the potential for effective practice and outcomes.

## Background

Scholars commonly acknowledge inconsistent and sparse reporting about the design and implementation of complex interventions within the published literature [1-3]. Complex interventions (also referred to as multiple interventions) deliberately apply coordinated and interconnected intervention strategies, which are targeted at multiple levels of a system [4]. Variable and limited reporting of complex interventions compromises the ability to answer questions about how and why interventions work through systematic assessment across multiple studies [3]. In turn, limited evidence-based guidance is available to inform the efforts of those responsible for the design and implementation of interventions, and the gap remains between research and practice.

The momentum within the last five years to identify promising practices in many fields [5-7] increases the urgency and relevance of understanding how and why interventions work. However, complex community health programs involve a set of highly complex processes [8-10]. It has been argued that much of the research on these programs has treated the complex interactions among intervention elements and between intervention components and the external context as a 'black box' [4,11-14]. Of particular relevance to these programs are failures to either describe or take into account community involvement in the design stages of an intervention [8]; the dynamic, pervasive, and historical influences of inner and outer implementation contexts [12,14-17]; or pathways for change [13,14]. A comprehensive set of propositions to guide the extraction of evidence relevant to the planning, implementation, and evaluation of complex community health programs is missing.

Our research team was interested in applying a set of propositions that arose out of a multiple intervention framework to examine reports on community health interventions [4]. To this end, we present a set of propositions that reflects best practices for intervention design, implementation, and evaluation for multiple interventions in community health, and we conduct a preliminary assessment of information reported in the published literature that corresponds to the propositions.

### Propositions for the design, implementation and evaluation of community health interventions

The initial sources for propositions were primary studies and a series of systematic and integrative reviews of many large-scale multiple intervention programs in community health (*e.g*., in fields of tobacco control, heart health, injury prevention, HIV/AIDS, workplace health) [8,10,18-24]. By multiple interventions, we mean multi-level and multi-strategy interventions [4]. Common to many of these were notable failures of well-designed research studies to achieve expected outcomes. Authors of these reviews have elaborated reasons why some multiple intervention programs may not have had their intended impact. Insights for propositions include researchers' reflections on the failure of their multiple intervention effectiveness studies to yield hypothesized outcomes, and reviews of community trials elaborating reasons why some multiple interventions programs have not demonstrated their intended impact [8,10,22,23,25,26]. The predominant and recurring reasons for multiple intervention research failures are addressed in the initial set of propositions for how and why interventions contribute to positive outcomes.

**Figure 1:**
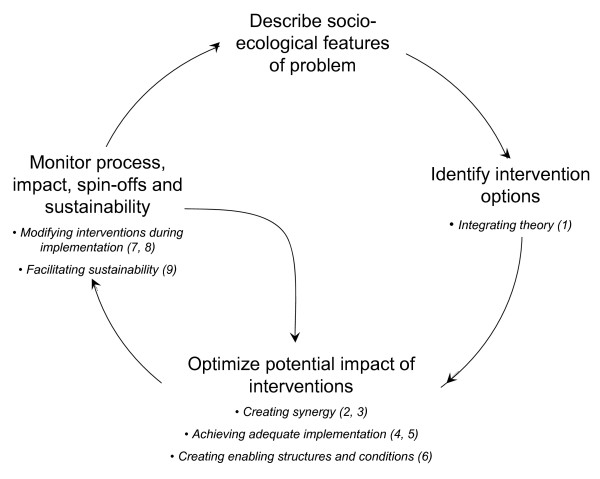
**Multiple Interventions Program Framework**. (adapted from Edwards, Mill & Kothari, 2004, reproduced with permission).

The propositions arise from and are organized within a multiple interventions program framework (see Figure 1 and Table 1). The framework is based on social ecological principles and supported by theoretical and empirical literature describing the design, implementation, and evaluation of multiple intervention programs [8-10,18-21,25-29]. The framework has four main elements, and several processes within these elements. The propositions address some of the common reasons reported to explain failures in multiple intervention research.

**Table 1:** Summary of propositions for multiple interventions in community health

**#**	**PROPOSITIONS**
**Identify intervention options**	

**Integrating theory**	
1	Relevant theories are integrated to contribute to a multi-level and multi-strategy intervention plan.

**Optimize potential impact of interventions**	

**Creating synergy**	
2	Combinations and sequences of interventions within and across levels of the system are used to create synergy.
3	Interventions create synergy through coordinating and integrating intervention efforts across sectors and jurisdictions.

**Achieving adequate implementation**	
4	Implementation of the interventions is sufficient to achieve population impacts.
5	The timing, the effort, and the features of the intervention strategies are tailored to the implementation context.

**Creating enabling structures and conditions**	
6	Relevant enabling structures and conditions at professional, organizational, community, and other system levels support the interventions.

**Monitor process, impact, spin-offs and sustainability**	

**Modifying interventions during implementation**	
7	Interventions are continuously adapted to the contextual environment (*e.g*., setting, leadership, structures, culture, etc.), while maintaining integrity with theoretical underpinnings.
8	Evaluation feedback is used to design interventions and to modify them throughout implementation.

**Facilitating sustainability**	
9	Sustainability – a focus on continuing and extending benefits of interventions – is addressed during planning, implementation, and maintenance phases of interventions.

## Methods

The preliminary assessment involved three main steps: selection of a sample of multiple intervention projects and publications, development of a data extraction tool, and data extraction from the publications.

### Selection of a sample of multiple intervention projects and publications

A first set of criteria was established to guide the selection of a pool of community-based multi-strategy and multi-level programs to use as case examples. The intent was not to be exhaustive, but to identify a set of programs that address a particular health issue that we anticipated might report details relevant to the propositions. The team decided reporting of such intervention features would most likely be represented in: a community-based primary prevention intervention program; a program that was well-resourced and evaluated, and thus represented a favorable opportunity for a pool of publications that potentially reported key intervention processes; and, a health issue that had been tackled using multiple intervention programs for a prolonged period, thus providing the maturation of ideas in the field.

In the last 30 years, community-based cardiovascular disease prevention programs have been conducted world-wide and their results have been abundantly published. The first pioneer community-based heart health program was the North Karelia Project in Finland, launched in 1971 [30]. Subsequent pioneering efforts included research and demonstration projects in the United States and Europe that included the Minnesota Heart Health Program, and Heartbeat Wales [9,31,32]. Although specific interventions varied across these projects, the general approach was similar. Community interventions were designed to reduce major modifiable risk factors in the general population and priority subgroups, and were implemented in various community settings to reach well-defined population groups. Interventions were theoretically sound and were informed by research in diverse fields such as individual behaviour change, diffusion of innovations, and organizational and community change. Combinations of interventions employed multiple strategies (*e.g*., media, education, policy) and targeted multiple layers of the social ecological system (*e.g*., individual, social networks, organizations, communities). Many of these exemplar community heart health programs were well-resourced relative to other preventive and public health programs, including large budgets for both process and outcome evaluations. Thus, community-based cardiovascular disease program studies were chosen as the case exemplar upon which to select publications to explore whether specific features of interventions as defined by the propositions were in fact described.

To guide the selection of a pool of published literature on community-based heart health programs, a second set of criteria was established. These included: studies representative of community-based heart health programs that were designed and recognized as exemplars of multiple intervention programs; studies deemed to be methodologically sound in an existing systematic review; and reports published in English. Selection of published articles meeting these criteria involved a two-step process. First, a search of the Effective Public Health Practice Project [33] was conducted to identify a systematic review of community-based heart health programs. The most recent found was by Dobbins and Beyers [25]. Dobbins and Beyers identified a pool of ten heart health programs deemed to be moderate or strong methodologically. From this pool, a subset of three projects was selected: the North Karelia Heart Health Project (1971–1992), Heartbeat Wales (1985–1990), and the Minnesota Heart Health Program (1980–1993), which were all well-resourced, extensively evaluated, and provided a pool of rigorous studies describing intervention effectiveness.

Second, a subset of primary publications identified in the Dobbins and Beyer's [25] systematic review was retrieved for each of the three programs. In total, four articles were retrieved and reviewed for the Minnesota Heart Health Program [34-37] and five articles for Heartbeat Wales [38-42]. For Heartbeat Wales, a technical report was also used because several of the publications referred to it for descriptions of the intervention [43]. The primary studies and detailed descriptions of the project design, implementation and evaluation for the North Karelia Project were retrieved from its book compilation [30].

### Development of a data extraction tool

The team was interested in identifying the types of intervention information reported, or not reported, in the published literature that corresponded with the identified best processes in the design, delivery, and evaluation of multiple intervention programs featured in the propositions. To enhance consistency, accuracy, and completeness of this extraction, a systematic method to extract the intervention information reported in the selected research studies was used. Existing intervention extraction forms [44,45] first were critiqued to determine their relevancy for extracting the types of intervention information corresponding to the propositions. These forms provided close-ended responses for various characteristics of interventions, but did not allow for the collection of information on the more complex intervention processes reflected in the propositions. Thus, the research team designed a data extraction tool that would guide the extraction of intervention information compatible with the propositions.

To this end, an open-ended format was used to extract verbatim text from the publications. Standard definitions for the proposition were developed (see Tables 2 through 7 in the results section), informed by key sources that described pertinent terms and concepts (*e.g*., sustainability, synergy) [46-51]. In order to enhance completeness and consistency of data extraction, examples were added to the definitions following an early review of data extraction (see below).

**Table 2:** Summary of data reported for integrating theory (proposition one)

**Operational Definition**	**Information Reported on Propositions**	**Illustrative Examples**
**Proposition one: Integration of relevant theories**		

Descriptions of theories, including any references regarding the relationships among the specific mid-range theories for the various dimensions of Multiple Intervention Programs including: the targets of change, channels, settings, and intervention strategies	A 'shopping list' of theories was reported	The 'program operated at the individual, group and community levels and encompassed a wide range of strategies stimulated by social learning theory, persuasive communications theory and models for the involvement of community leaders and institutions' [35:p.203]
	Most often, use of isolated theories was described for specific intervention design features	'The innovation of diffusion theory provided a central framework for the project team... the role of the project as a change agent was to promote the diffusion of the lifestyle innovations of quitting smoking and adopting low fat diets' [30: p.42] Organizational change theory was directed at improving the 'macro environment' while influencing individuals 'choices and opportunities to change' [38: p.8]
	Some reporting about the relationships among theoretical concepts through use of planning tool, such as a logic model	'The approaches described above are unified...to depict the behavioural/social model of community intervention found to be most relevant' [30: p.43]

**Table 3:** Summary of data reported for creating synergy (propositions two and three)

**Operational Definition**	**Information Reported on Propositions**	**Illustrative Examples**
**Proposition two: Combinations and sequencing/staging of interventions**		

Descriptions of the deliberate combination of interventions (implemented at the same time) and sequencing/staging of interventions (ordered in time) within and across levels of the system relative to their potential for enhanced synergistic and minimized antagonistic effects	Description regarding the combining and sequencing/staging of interventions at multiple levels of the system as an approach to optimizing overall program effectiveness and/or sustainability ranged from inferences to explicit details	'Staff training was implemented in work sites and churches to facilitate offering of health promotion programs such as quit smoking [30: p.203] The program consists of a 'complex set of projects and initiative which combine and interact in different ways to produce overall effect which is being measured through the outcome evaluation' [38: p.14] 'The aim is to promote synergism whereby each component reinforces the others' [43: p.89]
	Some referencing regarding the combining and sequencing/staging of interventions potentially attributable to both the anticipated positive outcomes, as well as explanation for shortfalls in expected outcomes.	The 'combination of mass communication and community organization.... was a valuable device for accelerating the diffusion of health innovation' [30: p.321] 'Intervention program may have focused on the wrong population segments or used the wrong mix of intervention components' [36: p.1391]
	More specific details were reported for the combining and sequencing/staging of interventions within levels of the system (such as interventions directed at the intrapersonal individual level), compared to across levels in the system (such as a combination of intrapersonal and policy level changes)	'In the two direct intervention schools, butter used on bread was replaced by soft margarine...These changes were also recommended for...meals at home...a nutritionist visited the homes of the children... Healthy diet was also discussed during school lessons. Parent gatherings, leaflets, posters, written recommendations, a project magazine, and the general mass media were used... Screening results were explained... A school nurse repeated the screening...and good advice and counseling to children...' [30: p.293] *Compared to*... 'With an effective political system, public health leaders can gain authority to strenuously exert influence over personal behaviours without arousing resistance.... this was accomplished through a blended approach which included both manipulation and empowerment [30: p.319]
	Reporting on the timing (sequential versus simultaneous) of interventions spanned from specific detail to general descriptions	'Actual screening programmes were often run simultaneously.' [30: p.97] 'Staggered entry of communities to intervention to allow for gradual development of the intervention program and strengthened the design through replication' [36: p.1384] 'The model Choice-Change-Champion process for health promotion' [was] constructed for 'idealized sequence of events' and intended to 'guide planning and priority setting'. [38: p.9] '...individuals are supported to move from stage one of having a 'choice' for lifestyle... through stage two of making 'changes' successfully... and stage three becoming a 'champion' for health at the local level which requires whereby individuals move from being a recipient to provider' [43: p.48]

**Proposition three: Coordinating and integrating intervention efforts**		

Descriptions of complementary interventions across sectors (*e.g*., health, education, recreation, labour, environment, housing, etc) and across jurisdictions (*i.e*., local/regional, provincial/state, federal/national).	Reporting on the importance and deliberate combining and sequencing/staging of interventions through use of multiple channels that crossed sectors and jurisdictions was both implicit and explicit	'The programme must be founded on intersectoral activity, community organization and grassroots participation.' [30: p.34] The development of advisory boards 'were made up of influential political business, health, and other leaders in the community and citizen task force' [35: p.202] 'The intervention comprises a wide range of locally organized projects together with centrally led initiatives...across all sectors of Welsh life, including the health and educational authorities, local and central government, commerce, industry, mass media, agricultural and voluntary sectors' [38: p.6]

**Table 4:** Summary of data reported for achieving adequate implementation (propositions four and five)

**Operational Definition**	**Information Reported on Propositions**	**Illustrative Examples**
**Proposition four: Adequate implementation**		

Quantitative descriptions of the intervention implementation, the amount and extent of engagement, include: 1. *duration *(time period); 2. *intensity *(depth of engagement such as passive receipt of information, interaction, or an environmental change); 3. *exposure *(total educational time, total minutes/hours/years of exposure); 4. *investment *(direct funding or in-kind contributions from various sources); 5. *reach *(*e.g*., total number of participants, proportion of population)	General information was often reported on the targeted audience rather than the reach (estimated numbers or proportions receiving intervention)	'Programme activities are usually simple and practical in order to facilitate their enactment by the widest spectrum of the community. Rather than the highly sophisticated services are generally simple basic services for a few people, simple basic services are generally provided for the largest possible stratum of the population' [30: p.48] 'All eighth graders enrolled in public schools' [34: p.219]
	Duration was generally reported for the overall program; total time for specific interventions was reported less frequently.	A TV series of 15 programmes called 'Key to Health' was broadcast during the 1984–85 school year.' [30: p.300] 'Systematic risk factor screening and education were conducted during the first 3 years of the intervention program' [35: p.202] 'first intervention – competition: took place over a 4 week community-wide competition' [34: p.219]
	Descriptions provided regarding the depth of engagement, including the passive receipt of information, to interaction, and environmental change	'The following list gives some idea of the extent to which print media were exploited during the five first years of the project (1972–77): local newspaper articles (877.000 column mm) 1509;...Health education leaflets (series of five) 278.000 copies...' [30: p.279] 'Activities were experiential – designed to require active participation' [37: p.1211] 'Activity was encouraged through a competition...role modeling...and environmental change' [34: p.219]
	Challenges to reporting cost and cost-benefits, as well as information regarding investment were described.	In evaluating the smoking component, cost-benefits were not calculated based on per-capita investment because a) cost of the smoking programme and its administration is 'impossible to estimate, or differentiate from usual operation', and b) the 'cost to some unites such as volunteers is not calculated' because of 'difficulty estimate it' [39: p.131] 'In 1990 the North Karelia Project employed nine full-time and eight part-time field office staff, who worked a total of over 18 000 hours that year' [30: p.66] 'The money to employ staff and finance the work has come from various sources' [39: p.72]

**Proposition five: Appropriate implementation**		

Qualitative descriptions regarding the quality of the intervention including: 1. fidelity (implementing all essential components of interventions as intended) 2. alignment with changing context (to ensure best fit); 3. implementing the most potent 'active ingredients'.	No explicit data reported regarding the quality of implementation	
	Descriptions regarding the quality of implementation were implicit, embedded in reporting of: 1. program features, such as priority setting or strategies undertaken to enhance quality implementation 2. explanations for problems with intervention fidelity relevant to explaining the results.	'One third (1/3) of the budget was dedicated to funding well-defined projects initiated locally that serve the objective of the program....' [38: p.17] 'Over its 20 years, the project has initiated or been otherwise involved in hundreds of training seminars. Although the nature of the seminars has changes, the focus has always been the discussion of practical tasks (derived for the objectives), action needed, and progress and feedback.' [30: p.278] 'After [the early years of the project ] it became both possible and necessary to introduce more specialized services to support the basic activities. These were prepared and tested by the project and implemented gradually'. [30: p.274]

**Table 5:** Summary of data reported for creating enabling structures and conditions (proposition six)

**Operational Definition**	**Information Reported on Propositions**	**Illustrative Examples**
**Proposition six: Enabling structures and conditions**		

Descriptions of the creation of structures (infrastructure) and conditions (processes and relationships) at system levels that support the design, implementation and/or evaluation of interventions, such as : media support; incentive grants; capacity building (for providers, organizations, communities); mechanisms for monitoring, evaluation, surveillance; networks; active citizen participation; opinion leader support.	Information regarding the deliberate creation of enabling structures and conditions was embedded in descriptions of intervention implementation.	'There was great stress placed on efforts to teach practical skills for change such as smoking cessation techniques and ways of buying and cooking healthier foods. For the latter, close co-operation with the local housewives' association has been proven invaluable, Activities have been coordinated to provide social support, expand options and availability (*i.e*., production and marketing of healthier foods), and ultimately to organize the community to function in a healthier mode' [30: p.40] 'Information gained from the community, clinical and youth baseline surveys about knowledge and lifestyles was shared in community meetings, with professional opinion leaders and published in easily understandable form for the local population...This served as a great force for...winning commitment from key decision makers, and motivating change among individuals and organizations.' [38: p.17]

**Table 6:** Summary of data reported for modification of interventions during implementation (propositions seven and eight)

**Operational Definition**	**Information Reported on Propositions**	**Illustrative Examples**
**Proposition seven: Adaptation to the contextual environment**		

Descriptions regarding the adjusting or tailoring of interventions to ongoing and unpredictable contextual changes, while maintaining theoretical underpinnings and integrity. Changes include such factors as: demographics, political priorities; organizational changes or priorities; economic environment; community events; network/coalition development, etc.	Authors described the importance of context and need for flexibility in intervention delivery	'Even when the framework of an intervention is well-defined...the actual implementation must be flexible enough to respond to changing community situations and to advantage of any fresh opportunities' [30: p.33]
	Details regarding what modifications were made to initial intervention implementation plans were vague, most often reported as part of the discussion for findings	'Project leaders and staff immersed themselves in the community and among the people, where they developed and adjusted programme activities according to the available local options and circumstances' [30: p.33]

**Proposition eight: Responsive to evaluation feedback**		

Descriptions regarding the collection and utilization of information about the process of intervention implementation, intervention outcomes (preliminary or later stage), or broader trends on risk factors or conditions, demographics, morbidity and mortality, etc.	Importance of process evaluation described as a tool for improving programs.	'Process evaluation '...is intended to identify features of a project which enhance or hinder its chances of success as the project develop' [38: p.14]
	Some description of how interventions were guided in response to preliminary evaluative information and population trends	'The project field office is actively involved with many aspects relating to process and formative evaluations. The health behaviour surveys have questions about the person's exposure to various intervention activities, which provides immediate feedback. The health education materials and media campaigns rely heavily on the result of the monitoring' [30: p.71] 'The 1987 population survey found that the decrease in population cholesterol means had leveled off. Novel and intensified activities began in North Karelia and across the country, coinciding with new national cholesterol guidelines' [30: p.108]
	Reporting on formative evaluation as *post hoc *activities in an attempt to explicate why expected outcomes were or were not achieved.	'There was suggestive evidence, however, that innovative modification in format could lead to renewed interest in contests' [35: p.204]

**Table 7:** Summary of data reported for facilitating sustainability (proposition nine)

**Operational Definition**	**Information Reported on Propositions**	**Illustrative Examples**
**Proposition nine: Sustainability**		

Discussion regarding the continuation or extension of the issue, program, partnerships, benefits, etc. Includes planning at the outset	Reporting on the notion of sustainability at the outset of the project	'In principle, a community-based project can vary from a relatively restricted academic study, or local effort, to a major programme with strong nationwide involvement. The North Karelia Project definitely falls into the latter category. At the very onset the national health authorities decided that the North Karelia Project would be a pilot for all Finland.' [30: p.51]
	Description of conditions and supports in place that would facilitate sustainability such as finances, partnerships, and previous experience	'The fact that the project director represented North Karelia in the National Parliament from 1987–1991 was important in this respect. The cooperation of the local health services and health personnel has guaranteed a firm foundation for the project activities. Numerous community organizations have also contributed greatly over the years. Because project activities have been integrated into the existing health services and broad community participation has been a key feature, the overall costs of the programme have been kept modest.' [30: pp.71–72] 'The project has arranged numerous competitions in collaboration with the food-industry, the media, schools, sports clubs, voluntary organizations etc. over the past twenty years' [30: p.287] 'During the project several of its leading members have been active in various health and health research policy functions' [30: p.287]
	Descriptions of sustainability evidenced in outcomes of the program such as policy change and extension of the issue illustrated by the role of projects as a catalyst for other jurisdictions	'The creation by Secretary of State for Wales of The Welch Health Promotion Authority with clear brief to sustain and support the program provide longer possibilities for Heartbeat Wales' [38: p.17] This 'new administrative arrangements...ensure the future and.. support the complementary initiatives on health promotion for young people and sensible drinking' [40: p.346] 'The project became associated with healthy public policy in may ways, by contributing to anti-smoking legislation, for instance.' [30: p.43] 'The project has been a major and diverse contributor to many policy decisions on the national and local levels' [39: pp.71–72] 'The North Karelia Project has itself been a model for imitation and acceleration of similar activities around the world [30: p.322] 'It was considered worthwhile for the project to continue operating beyond the initial five-year period, but at the same time to expand activities to contribute to national developments. So while North Karelia continued to be an active demonstration area the project evolved a national dimension to its activities' [30: p.360]

### Data extraction from the publications

Pairs from the research team were assigned to one of the three heart health projects. Information from the studies was first extracted independently, and then the pairs for each project compared results to identify any patterns of discrepancies. Throughout the process, all issues and questions related to the data extraction were synthesized by a third party. Early on, examples were added to the definitions of the propositions to increase consistency of information extracted with respect to content and level of detail. Through discussion within pairs and across the research team, consensus was reached on information pertinent to the propositions, and each pair consolidated the information onto one form for each project. The consolidated form containing the consensus decisions from each pair was then used to compare patterns across the full set of articles. All members of the research team participated in the process to identify trends and issues related to reporting on relevant intervention processes. These trends and issues are described in the next section.

## Results

Results are reported for each proposition in order from one through nine, and grouped according to the themes shown in the multiple interventions program framework (Figure 1). For each proposition, results are briefly described in the text. These descriptions are accompanied by a table that includes the operational definition for the proposition, findings related to reporting on the proposition, and illustrative verbatim examples from one or more of the projects.

### Integrating theory (proposition one)

Information regarding the use of theories was most often presented as a list, with limited description of the complementary or unifying connections among the theories in the design of the interventions. Commonly, intervention programs projected changes at multiple socio-ecological levels, such as individual behaviour changes, in addition to macro-environmental changes. However, while theories were used for interventions targeting various levels of the system, the integration of multiple theories was generally implicit and simply reflected in the anticipated outputs. Although less common, the use of several theories was made more explicit through description of the use of a program planning tool, such as a logic model (Table 2).

### Creating synergy (propositions two and three)

General references were frequently made regarding the rationale for combining, sequencing, and staging interventions as an approach to optimizing overall program effectiveness and/or sustainability. In particular, references to this were most often found in proposed explanations for shortfalls in expected outcomes. However, specific details regarding how intervention strategies were combined, sequenced, or staged across levels, as well as across sectors and jurisdictions, were usually absent. Thus, insufficient information was provided to understand potential synergies that may have arisen from coordinating interventions across sectors and jurisdictions. In contrast, more specific details were reported for the combining, sequencing, and staging of interventions within levels of the system (*i.e*., a series of interventions directed at the intrapersonal level) (Table 3).

### Achieving adequate implementation (propositions four and five)

Proposition four specifically considers the quantitative aspects of implementation. Information reported ranged from general statements to specific details. Although the population subgroups targeted by the intervention were often clearly identified, information regarding the estimated reach of the intervention was generally non-specific. The amount of time for specific intervention strategies and the overall program tended to be reported in time periods such as weeks, months or years. Information regarding specific exposure times for interventions tended to be unavailable. The intensity of interventions was provided in some reports, with authors describing strategies that included the passive receipt of information, interaction, and/or environmental changes. A description of investment levels is also a marker of the intensity of an intervention strategy. However, investment descriptions were quite variable, ranging from no information to general information on investment of human and financial resources. In addition, challenges to reporting costs and benefits were often acknowledged.

Proposition five considers the quality of implementation, represented by qualitative descriptions of the intervention. Reporting regarding the quality of the implementation was primarily implicit (Table 4).

### Creating enabling structures and conditions (proposition six)

Reporting of information relative to the deliberate creation of structures and conditions was limited and generally implicit, often embedded in the details of intervention implementation (Table 5).

### Modifying interventions during implementation (propositions seven and sight)

Although authors acknowledged the importance of flexibility in intervention delivery, information regarding adaptations to environmental circumstances was vague. Reference to context was often in discussion sections of studies, and provided as a partial explanation for unintended or unexpected outcomes. There was minimal description regarding the modification of interventions in response to information gained from process/formative evaluation, outcomes, or population trends – the core of proposition eight. Again, authors acknowledged the significance of process/formative evaluation in informing intervention implementation, with some examples to illustrate how interventions were guided in response to information gathered. At other times, in the summative evaluation, reporting focused on using process evaluation results to explain why expected outcomes were or were not achieved, rather than how the process evaluation results did or did not shape the interventions during implementation. Suggestions for improved program success, based on information gained from formative evaluations, were noted in some discussions (Table 6).

### Facilitating sustainability (proposition nine)

Reporting on elements regarding the intention to facilitate sustainability of multiple intervention benefits was also variable. Authors made reference to the notion of sustainability at the onset of projects and described the conditions and supports that were in place to facilitate continued and extended benefits. Elements of sustainability represented in program outcomes were also described in some detail. In other examples, reporting only focused on sustainability of the program during the initial research phase of program implementation and discussed the desirability of continuing the program beyond the research phase (Table 7).

## Discussion

The primary purpose of this paper was to conduct a preliminary assessment of information reported in published literature on 'best' processes for multiple interventions in community health. It is only with this information that questions of how and why interventions work can be studied in systematic reviews and other synthesis methods (*e.g*., realist synthesis). The best processes were a set of propositions that arise from and were organized within a multiple interventions program framework. Community-based heart health exemplars were used as case examples.

Although some information was reported for each of the nine propositions, there was considerable variability in the quantity and specificity of information provided, and in the explicit nature of this information across studies. Several possible explanations may account for the insufficient reporting of implementation information. Authors are bound by word count restrictions in journal articles, and consequently, process details such as program reach might be excluded in favour of reporting methods and outcomes [3]. Reporting practices reflect what traditionally has been viewed as important in intervention research. There is emphasis on reporting to prove the worth of interventions over reporting to improve community health interventions. This follows from the emphasis on answering questions of attribution (does a program lead to the intended outcomes?), rather than questions of adaptation (how does a dynamic program respond to changing community readiness, shifting community capacity, and policy windows that suddenly open?) [16,52].

An alternative explanation is that researchers are not attending to the processes identified in the propositions when they design multiple intervention programs. Following these propositions requires a transdisciplinary approach to integrating theory, implementation models that allow for contextual adaptation and feedback processes, and mixed methods designs that guide the integrative analysis of quantitative and qualitative findings. These all bring into question some of the fundamental principles that have long been espoused for community health intervention research, including issues of fidelity, the use of standardized interventions, the need to adhere to predictive theory, and the importance of following underlying research paradigms. When coupled with the challenges of operationalizing a complex community health research study that is time- and resource-limited, it is perhaps not surprising that the propositions were unevenly and weakly addressed.

It would be premature to generalize these results to other programs. The three multiple intervention programs (the North Karelia Project, Heartbeat Wales, and the Minnesota Heart Health Program) selected for this study were implemented between 1971 and 1993, and represented the 'crème de la crème' of heart health programs in terms of study resources and design. In particular, the North Karelia project continues to receive considerable attention due to the impressive outcomes achieved [17]. We think it would be useful to apply the data extraction tool developed by our team to some of the more contemporary multiple intervention programs targeting chronic illness. Our findings would provide a useful basis of comparison to determine whether or not there has been an improvement over the past decade in the reporting of information that is pertinent to the propositions. Before embarking on this step, it would be helpful to have further input on the data extraction tool, particularly from those who are involved in the development of new approaches to extract data on the processes of complex interventions with the Cochrane initiative [3].

## Conclusion

Study findings suggest that limited reporting on intervention processes is a weak link in published research on multiple intervention programs in community health. Insufficient reporting prevents the systematic study of processes contributing to health outcomes across studies. In turn, this prevents the development and implementation of evidence-based practice guidelines. Based on the findings, and recognizing the preliminary status of the work, we offer two promising directions.

First, it is clear that a standard tool is needed to guide systematic reporting of multiple intervention programs. Such a tool could inform both the design of such research, as well as ensure that important information is available to readers of this literature and to inform systematic analyses across studies. In addition, a research tool that describes best processes for interventions could benefit practitioners who are responsible for program design, delivery, and evaluation.

Second, the reasons for limited reporting on intervention processes need to be understood. Some issues to explore include the influence of publication policies for relevant journals, and the types of research questions and processes that are used.

It is through a more concerted effort to describe and understand the black box processes of multiple interventions programs that we will move this field of research and practice forward. It is our contention that a shift to more inclusive reporting of intervention processes would help lead to a better understanding of successful or unsuccessful features of multi-strategy and multi-level interventions, and thereby improve the potential for effective practice and outcomes.

## Competing interests

The authors declare that they have no competing interests.

## Authors' contributions

BR conceived of the study, managed the project, and was the lead writer. JM led development of the data extraction tool. OM led the description of results. NE conceived of the multiple interventions framework and co-developed the propositions with BR. All authors contributed substantively to the operational definitions, data extraction, and writing. All authors have read and approved the final manuscript.
